# Vitamin D and gut microbiota in thyroid diseases: mechanisms and therapeutic potential

**DOI:** 10.3389/fimmu.2026.1878820

**Published:** 2026-09-17

**Authors:** Mei Jiao, Xiaodan Zhai, Zheng Jia, Boshen Gong, Ruofan Shen, Ying Liu, Dianbao Zhang, Zhongyan Shan, Chuyuan Wang

**Affiliations:** 1 Department of Endocrinology and Metabolism, The Institute of Endocrinology, National Health Commission (NHC) Key Laboratory of Diagnosis and Treatment of Thyroid Diseases, The First Hospital of China Medical University, Shenyang, China; 2 Department of Endocrinology, Shengjing Hospital of China Medical University, Shenyang, Liaoning, China; 3 Department of Stem Cells and Regenerative Medicine, Key Laboratory of Cell Biology, National Health Commission of China, and Key Laboratory of Medical Cell Biology, Ministry of Education of China, China Medical University, Shenyang, China; 4 School of Life Sciences, Liaoning University, Shenyang, China

**Keywords:** autoimmune thyroiditis, gut microbiota, short-chain fatty acids, thyroid-gut axis, vitamin D

## Abstract

Thyroid diseases have become the most common endocrine disorders globally, including hyperthyroidism, hypothyroidism, thyroid nodules (TN), and thyroid cancer (TC). Traditionally, the pathogenesis was attributed to autoimmune dysfunction, iodine metabolism imbalance, and genetic-environmental interactions. Recently, research suggests that vitamin D deficiency and gut microbiota dysbiosis are prevalent in patients with thyroid disorders, particularly in autoimmune thyroid disease (AITD). Vitamin D, as well as gut microbiota and its metabolites, such as short-chain fatty acids and bile acids, jointly regulate innate and adaptive immunity. They also mitigate intestinal leakage by maintaining intestinal barrier integrity, thereby influencing the development and progression of thyroid diseases. Furthermore, studies confirm the bidirectional interaction between vitamin D and the gut microbiota. Vitamin D supplementation increases the abundance of beneficial bacteria and regulates their metabolites. While beneficial bacteria and their metabolites, such as butyrate and bile acids, promote vitamin D receptor (VDR) expression and enhance vitamin D bioavailability. In this review, we integrate existing current basic and clinical evidence to systematically dissect the individual functions and reciprocal crosstalk of vitamin D and gut microbiota in thyroid diseases. Furthermore, we put forward a combined intervention of vitamin D and gut microbiota, which may serve as a viable therapeutic strategy for thyroid disease.

## 1 Introduction

As one of the most prevalent endocrine and metabolic disorders, thyroid diseases have evolved into a major global public health concern, with their incidence rates rising steadily worldwide. The clinical spectrum of thyroid diseases is remarkably diverse, encompassing hyperthyroidism, hypothyroidism, thyroid nodules (TN), and thyroid cancer (TC). Beyond impairing thyroid function, these conditions often exert far-reaching systemic effects, affecting multiple organ systems, including the cardio-vascular, neurological, and reproductive systems (1, 2).

For decades, research on the causes and mechanisms of thyroid disease has primarily focused on autoimmune dysregulation, iodine metabolism imbalances, and gene environmental interactions. Autoimmune dysfunction is central to the pathogenesis of autoimmune thyroid disease (AITD). Dysregulation of CD4^+^ T-lymphocyte subsets, such as expansion of pro-inflammatory Th17 cells and reduction of immunosuppressive Treg cells, results in Th17/Treg imbalance and further induces thyroid tissue injury (3, 4). Iodine deficiency triggers abundant reactive oxygen species production and insufficient substrate for thyroid hormone synthesis, which results in thyroid nodules and hypothyroidism; excessive iodine intake increases thyroid autoantibody levels and thus induces AITD (5, 6). Meanwhile, genetic susceptibility and environmental factors, such as infections, stress, and certain medications, work together to promote the development and progression of thyroid diseases (7).

In recent years, vitamin D and gut microbiota have garnered widespread attention as emerging factors, offering novel perspectives on understanding thyroid disorders. Research indicates that vitamin D deficiency is common among patients with thyroid disease. And it’s inversely correlated with thyroid autoantibody levels, such as thyroid peroxidase antibody (TPOAb) and thyroglobulin antibody (TGAb) (8). Through the vitamin D receptor (VDR), vitamin D not only participates in the regulation of innate and adaptive immunity but also maintains the function of the intestinal barrier, thereby affecting thyroid homeostasis (9–11). Multiple cross-sectional studies have demonstrated gut microbiota dysbiosis in patients with thyroid diseases, predominantly characterized by reduced abundance of beneficial commensal bacteria (12). Beneficial microbes and their metabolites, such as butyrate and bile acids, modulate immune responses and maintain intestinal barrier integrity through the gut-thyroid axis (13). These molecular events further contribute to the regulation of thyroid function. Importantly, a mutual regulation also exists between vitamin D and the gut microbiota. Beneficial bacteria and their metabolites, such as butyrate and bile acids, appear to act as key mediators in this process (14, 15). Nevertheless, most existing studies have separately investigated the effects of either vitamin D or gut microbiota on thyroid disorders, while the synergistic actions of these two factors in thyroid diseases remain to be systematically summarized. Addressing this research gap and integrating the above-mentioned correlative evidence, we hypothesize that a coordinated vitamin D-gut microbiota-thyroid regulatory axis may exist. Acting through the core hubs of immune function and intestinal barrier homeostasis, vitamin D and gut microbiota may jointly contribute to the initiation and progression of thyroid diseases.

This review summarizes clinical evidence on the roles of vitamin D and gut microbiota in thyroid diseases, investigates the mechanisms of their interaction, and puts forward a new concept of their coordinated regulation in thyroid pathophysiology.

## 2 Vitamin D deficiency and its roles in thyroid diseases

### 2.1 Metabolism and pleiotropic physiological functions of vitamin D

Vitamin D exists in two major forms: vitamin D_2_ derived from plant sources, and vitamin D_3_, which is synthesized in the skin and obtained from animal-based foods (16, 17). Vitamin D_3_ is the most predominant form of vitamin D in the human body. Vitamin D_3_ is first converted in the liver by 25-hydroxylase into 25(OH)D_3_, and then activated in the kidneys or extrarenal tissues by 1α-hydroxylase to form 1,25(OH)_2_D_3_. The active form exerts its biological effects by binding to the VDR (18, 19). VDR is widely distributed across various tissues and participates in multiple physiological processes, including calcium-phosphate metabolism, immune regulation, and cell differentiation (20, 21).

The functions of vitamin D can be classified into classical and non-classical roles (**Figure 1**). Its classical action mainly involves regulating calcium-phosphate balance and supporting bone health (22). Non-classical functions extend to immune modulation, cell proliferation and differentiation, as well as cardiovascular and neurological regulation (22, 23). Vitamin D modulates cell-cycle progression and physiological cell differentiation and maturation, while suppressing aberrant cell proliferation. In the cardiovascular system, vitamin D preserves vascular endothelial barrier integrity and alleviates endothelial inflammatory injury (24). Its regulatory effects on the nervous system are achieved by attenuating neuronal oxidative damage and inhibiting neuroinflammatory signaling pathways. All these non-canonical biological functions are mediated through two distinct pathways (25). Vitamin D exerts its regulatory activities either by directly binding to VDR expressed on target tissue cells, or indirectly by modulating immune cells to trigger relevant physiological responses (26). Immune cells express VDR, allowing vitamin D to play a significant regulatory role in autoimmune disorders (27). Vitamin D modulates both innate and adaptive immunity. In innate immunity, it promotes monocyte differentiation into macrophages, stimulates the production of antimicrobial peptides, inhibits the activation and maturation of dendritic cells, and strengthens tight junctions between intestinal epithelial cells (28–30). Regarding adaptive immunity, vitamin D primarily influences B cells and T cells. It can suppress autoantibody production by B cells, inhibit the differentiation of Th1 and Th17 cells while promoting Th2 and Treg cell development, and help balance cytokine profiles, thereby exerting anti-inflammatory effects (31–33).

**Figure 1:**
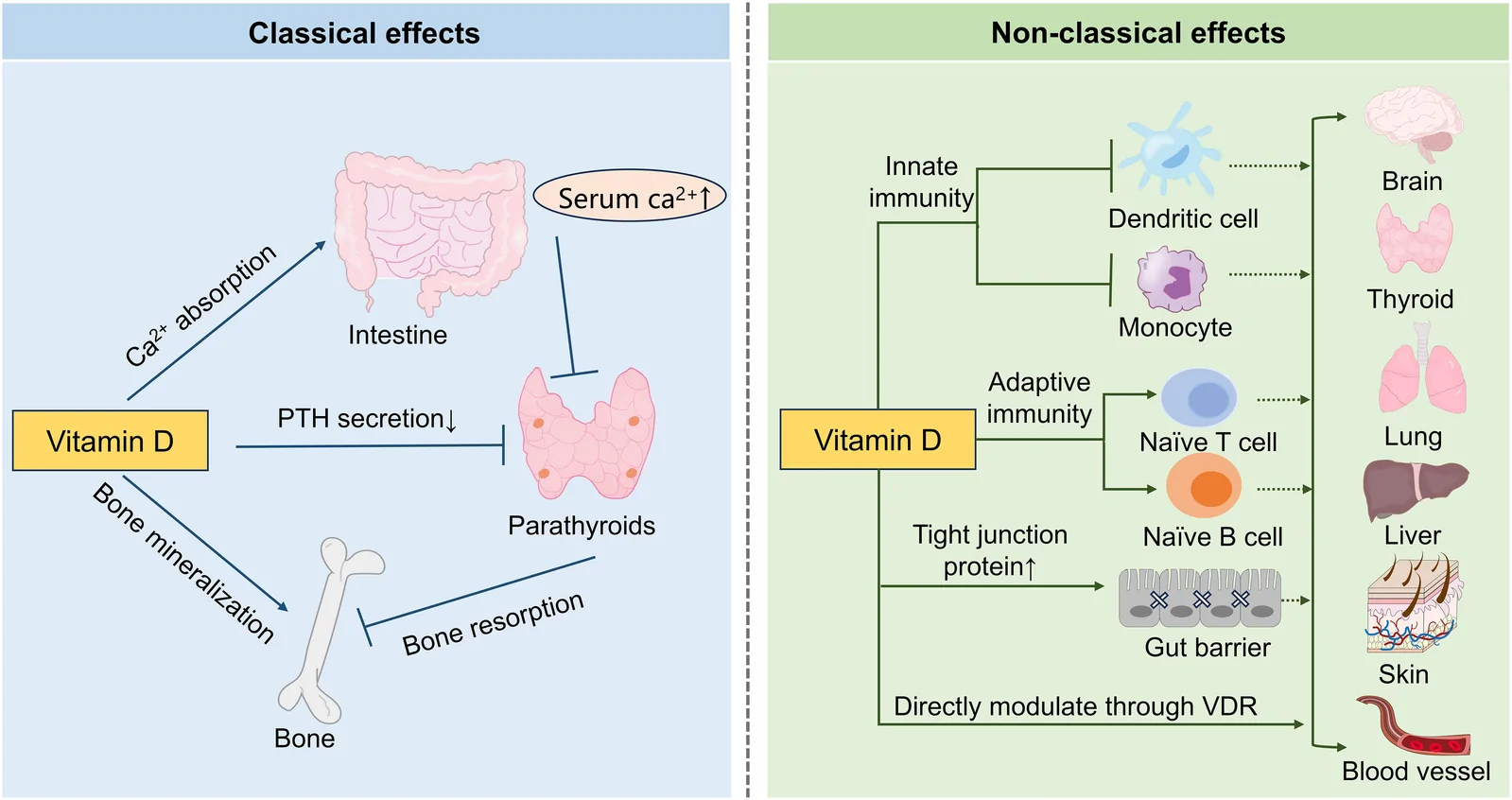
Schematic diagram of classical versus non-classical actions of vitamin D. The classical effects focus on calcium homeostasis and bone metabolism, including regulation of intestinal calcium uptake, parathyroid hormone secretion, and bone remodeling. In its non-classical effects, vitamin D modulates a wide range of vital tissues and organs, such as the brain, thyroid, lung, liver, skin, and blood vessels. Immunomodulation and intestinal barrier protection exert crucial regulatory functions herein. PTH: parathyroid hormone; ↑: increase; ↓: decrease.

### 2.2 Vitamin D deficiency in AITD

We summarized clinical studies on vitamin D levels in AITD patients published over the past decade reveals some inconsistent findings, which may be related to factors such as sample size, research methodology, and population characteristics (**Table 1**).

**Table 1:** The levels of thyroid autoantibodies and vitamin D in AITD.

Study groups	Changes in vitamin D	Correlation with antibody titers	Limitations	Citation
112 euthyroid HT patients 178 HCs	HT patients: significantly lower	Vitamin D and TPOAb: negative correlation	Single-center study; small sample size of HT patients; only female cohort	(36)
114 HT patients (subgrouped by thyroid function)	HT patients: significantly lower	Vitamin D and TPOAb: negative correlation	Small sample size; variable HT diagnostic criteria	(37)
179 HT patients	25 (OH) D increased after vitamin D supplementation	TPOAb/TSH ↓; FT3/FT4 ↑	Cholecalciferol only; small sample size; short follow-up	(38)
42 HT patients	25 (OH) D increased after vitamin D supplementation	TGAb vs. baseline ↓; no significant change in TPOAb vs. placebo	Small sample size; only females; short follow-up	(34)
30 HT 30 GD 20 HCs	HT/GD vs. controls: significantly lower	Vitamin D and TGAb/TPOAb in HT: negative correlation	Small sample size	(8)
105 HT patients 80 HCs (analyzed by sex)	female HT vs. controls: higher; no difference in males	In males vitamin D and TPOAb: positive correlation; no correlation with TgAb or TSH	Controls not tested for thyroid autoimmunity	(35)
218 HT patients (180 Female, 38 Male)	186 HT patients: significantly lower	Vitamin D and TPOAb: negative correlation; 20% decrease in anti-TPO after supplementation	No control group; no blinding; no histological confirmation of HT	(39)
40 GD patients (22 on ATD, 18 on ATD + Vit D), 37 HCs	GD vs. HCs: significantly lower	No difference in TRAb between GD patients with or without vitamin D supplementation	Small sample size; short follow-up	(40)
GD patients (grouped by Vit D status) HCs	GD vs. HCs: lower	Serum 25(OH)D negatively correlated with TSHAb	Small sample size; short follow-up	(41)
210 GD patients with vitamin D deficiency	Level increased from 10.6 to 25.7 ng/mL in the supplemented group; 11.6 ng/mL in the non-supplemented group.	Vitamin D level and TBII titer at ATD discontinuation were independent predictors of relapse (weak negative correlation)	Non-randomized (patient choice), selection bias	(42)
292 GD patients 2305 HCs	GD vs. HCs: lower (55.0 vs. 87.2 nmol/L)	No association between vitamin D and TRAb titer, GO, or relapse	No association conclusion limited to baseline; cross-sectional study	(43)
51 GD 61 mild HT 63 severe HT 51 HCs	HT vs. HCs: lower; no significant difference in GD	No association between vitamin D and thyroid autoantibodies in AITD patients	Small sample size; short follow-up	(44)

AITD, Autoimmune Thyroid Disease; HT, Hashimoto’s Thyroiditis; GD, Graves’ Disease; HCs, Healthy Controls; TPOAb, Thyroid Peroxidase Antibody; TGAb, Thyroglobulin Antibody; TRAb, TSH Receptor Antibody; TSHAb, Thyroid-Stimulating Hormone Antibody; TBII, TSH-Binding Inhibitory Immunoglobulin; ATD, Antithyroid Drugs; GO, Graves’ Orbitopathy; Vit D, Vitamin D.

↑ indicates increase bacterial abundances; ↓ indicates decrease bacterial abundances.

In the majority of clinical studies on Hashimoto’s thyroiditis (HT), patients exhibit significantly lower vitamin D levels than healthy controls. Thyroid autoantibodies (TPOAb, TGAb) are negatively correlated with vitamin D status. Nevertheless, certain clinical studies have reached contradictory conclusions. For example, the studies by Chahardoli R and Yasmeh J reported no correlation or even a positive association between thyroid autoantibodies and vitamin D levels (34, 35). In most studies on Graves’ disease (GD), vitamin D levels in GD patients are generally below the normal baseline, but autoantibody levels often show no significant correlation with vitamin D status. However, most of these findings come from small-scale studies with short follow-up periods. Future research should involve larger-scale, multicenter clinical trials with a minimum follow-up period of 12 months to further validate these observations.

### 2.3 Vitamin D deficiency in TN

Several clinical studies have demonstrated reduced serum vitamin D concentrations among patients with TN, suggesting a potential link between vitamin D status and TN risk. For example, a cross-sectional investigation reported that markedly lower vitamin D levels were observed in TN patients relative to control group (45). Nathan et al. also reported vitamin D deficiency in patients with TN (46). However, this study was limited by its small sample size and absence of a healthy control group. In contrast, a recent large-scale prospective cohort study based on the UK Biobank database clearly indicated a negative correlation between TN risk and vitamin D levels. It proposed a threshold vitamin D level of 60.7 nmol/L for the development of TN (47). Fan et al. surveyed 875 centenarians and found a negative association between TN incidence and vitamin D levels (48). Nevertheless, the study only enrolled elderly participants, which may introduce selection bias into its conclusions.

Although most studies support an association between vitamin D deficiency and TN, the relationship with clinical prognosis remains controversial. A retrospective study showed that vitamin D levels were not associated with TN size and suggested that vitamin D levels could not serve as a prognostic indicator for TN (49). Moreover, existing studies are mostly observational and cannot rule out some confounding factors. Some studies also suffer from insufficient sample sizes and limited populations. Future large-scale, multicenter, prospective interventional studies are needed to clarify the role of vitamin D supplementation in the prevention and treatment of TN.

### 2.4 Vitamin D deficiency in TC

Most studies suggest that vitamin D deficiency is a risk factor for TC, especially for differentiated thyroid cancer (DTC), and is associated with worse prognostic features. Two meta-analyses provide evidence-based medical support for this association. Yang L et al. found that vitamin D levels in TC were significantly lower than those in healthy individuals, and that vitamin D deficiency was significantly associated with an increased risk of thyroid cancer (50). Another meta-analysis indicated that for every 10 ng/ml decrease in 25(OH)D, the risk of TC increased by 6% (51). Furthermore, some researchers have explored the underlying mechanisms through basic experiments. VDR inhibits DTC cell proliferation and promotes differentiation by regulating the E-cadherin/β-catenin complex (52). However, the current conclusions remain controversial. One Mendelian randomization study found no causal relationship between vitamin D levels and thyroid cancer risk (53). Another systematic review also showed that VDR gene polymorphisms are not associated with increased susceptibility to differentiated TC (54). The inconsistency in these clinical findings may be due to differences in research methods, sample sizes, and TC stage.

### 2.5 Targeted vitamin D therapy and its clinical evidence in thyroid diseases

Observational studies have illustrated a high incidence of vitamin D deficiency in patients with thyroid disorders. However, these studies merely demonstrate statistical correlation instead of verifying causal interaction between vitamin D status and thyroid pathogenesis. To clarify whether vitamin D supplementation ameliorates the progression of AITD and to standardize its clinical dosage and administration, multiple randomized controlled trials (RCTs) have been conducted in AITD populations. Accumulated evidence from systematic reviews and meta-analyses of RCTs has validated that vitamin D supplementation markedly reduces circulating TPOAb and TgAb titers (55, 56). Further meta-analytic evidence indicates that continuous vitamin D intervention lasting no less than three months can alleviate thyroid autoimmunity, independent of baseline thyroid function (57, 58). Currently, no unified guideline has established standardized vitamin D supplementation protocols specifically for thyroid disease patients. Serum 25(OH)D is recognized as the critical biomarker for evaluating vitamin D nutritional status. Generally, serum 25(OH)D levels below 20 ng/mL indicate vitamin D deficiency, while levels between 21 and 29 ng/mL suggest relative insufficiency (59). Existing clinical evidence recommends maintaining serum 25(OH)D concentrations at 30–45 ng/mL for AITD patients (60). Nevertheless, the optimal supplementation strategy for thyroid diseases remains undefined, which requires further validation through large-scale clinical trials.

Supplementation with fixed-dose vitamin D yields substantial heterogeneity in therapeutic effects among patients with thyroid disorders. Carlberg and Haq put forward the concept of the “individual vitamin D response index”. This classification stratifies individual responses to vitamin D intervention according to chromatin accessibility and the transcriptional activity of VDR target genes in peripheral immune cells (61). This index differs distinctly from serum 25(OH)D measurement. Solely relying on circulating 25(OH)D levels to guide vitamin D supplementation therefore fails to fully stratify patients and optimize intervention strategies. Under these circumstances, precision vitamin D nutritional intervention has gradually become an innovative clinical direction. Combined with multi-omics techniques and computational analysis, this personalized strategy formulates targeted supplementation regimens based on individual genetic characteristics, gut microbial composition, disease phenotypes, and environmental factors (62, 63). Recent advances in nutrigenomics have identified specific single nucleotide polymorphisms (SNPs) in key genes governing vitamin D metabolism, namely CYP2R1, CYP27B1, GC (encoding vitamin D-binding protein), and VDR (64). These genetic variants alter the efficiency of vitamin D hydroxylation and receptor activation, thereby modulating individual responses to vitamin D supplementation. A retrospective study reported a high prevalence of VDR FokI and ApaI polymorphisms among patients with HT (65). Specifically, lower circulating vitamin D concentrations have been associated with the homozygous genotype at the FokI locus of the VDR gene. Beyond genetic variations, the gut microbiota represents another key modulator of the *in vivo* effects of vitamin D. By regulating vitamin D bioavailability and VDR expression levels, the gut microbiota has been incorporated as a critical reference dimension in precision nutritional intervention strategies (66).

Nevertheless, precision nutritional vitamin D intervention for thyroid diseases remains at an exploratory stage and has not yet been translated into routine clinical practice (61). Despite its promising prospects, large-scale, multicenter interventional studies are warranted to bridge the gap between research evidence and clinical application in the future.

## 3 Alteration of gut microbiota in thyroid diseases

### 3.1 Roles of the human gut microbiota

The human gut microbiota comprises bacteria, archaea, viruses, and eukaryotes, with bacteria primarily belonging to the phyla Firmicutes and Bacteroidetes (67, 68). The gut microbiota establishes a mutualistic symbiotic relationship with the host and extensively modulates host nutritional metabolism, immune responses, intestinal barrier function, and nervous system activities (69, 70). In terms of nutrient metabolism, the gut microbiota not only enhances protein and lipid metabolism but also synthesizes essential vitamins (71). For the nervous system, gut microbiota and their metabolites promote the activation of nerve cells and the expression of neurotrophic factors (72). In the process of maintaining intestinal homeostasis, gut microbiota function synergistically through multiple mechanisms. Commensal bacteria compete with pathogenic microorganisms for intestinal nutrients to suppress the colonization of harmful bacteria (73). Meanwhile, they participate in mucus layer formation to strengthen the physical intestinal barrier and upregulate tight junction protein expression, thereby reducing intestinal permeability. Furthermore, gut microbiota and their metabolites drive Treg cell differentiation and curtail the release of pro-inflammatory cytokines to regulate host immune responses (74–76). However, the gut microbiota is not a constant entity but a dynamic system. Studies show that host factors, including diet, exercise, medication, surgery, and stress, can alter the structure and function of the microbiota, leading to gut dysbiosis (77). This imbalance can subsequently affect immune balance and barrier function and is associated with various diseases, including obesity, diabetes, inflammatory bowel disease, rheumatoid arthritis, and thyroid disorders.

### 3.2 Characteristics of gut microbiota in AITD

Research suggests that patients with AITD exhibit alterations in the composition and diversity of their gut microbiota. However, conclusions from clinical studies on the gut microbiota in AITD patients are inconsistent, likely due to variations in sample sizes, research methodologies, diet, and geographic regions (**Table 2**).

**Table 2:** Alterations in gut microbiota in AITD.

Study group	Changes in the gut microbiota	Limitations	Citation
16 HT 18 HCs	**HT group**: *Bacteroides fragilis*, and *Haemophilus parainfluenzae* ↑	Small sample size	(79)
31 HT 30 HCs	**HT group**: phylum level: Bacillota and Spirochaetota ↑	Small sample size; all-female cohort	(80)
25 HT 23 HCs	**HT group**: *Clostridia*_UCG-014, *Acidaminococcales*, *Oscillospirales*, and *Desulfovibrionales* ↑	Small sample size	(81)
27 HT 27 GD 16 HCs	**HT group**: *Proteobacteria* and *Actinomycetes*↑ **GD and HT groups**: *Erysipelotrichia*, *Cyanobacteria*, *Ruminococcus*_2 ↑ *Corynebacteridae, Megamonas* ↓	Single-center study; small sample size; geographical limitations	(82)
37 HT with euthyroidism 10.HT with thyroid dysfunction 42 HCs	**HCs group**: *Lachnoclostridium, Bilophila*, *Klebsiella*↑ **HT group with euthyroidism**: *Ralstonia*, *Fournierella*, *Megamonas* ↑ **HT group with thyroid dysfunction**: *Acetitomaculum*, *Shuttleworthia*, Flavobacteriaceae, Lachnospiraceae ↑	HT with thyroid dysfunction: difficult to collect; small sample size	(83)
20 AITD 30 HCs	**AITD group**: Phylum level: Firmicutes ↓ Bacteroidetes ↑ F/B↓ genus level: *Prevobacteria Akkermansia, Bifidobacteria, Lactobacillus*, *Clostridium* pras ↓	Small sample size	(84)
45 HT with euthyroidism 18 HT with hypothyroidism	**HT patients with euthyroidism**: Lachnospiraceae incertae *sedis*, *Lactonifactor*, *Alistipes*, *Subdoligranulum* ↑ **HT Patients with hypothyroidism**: *Phascolarctobacterium* ↑	Single-center study; small sample size	(85)
9 HT 9 GD 11 HCs	**GD group**: *Fusobacterium, Fusobacterium, and Sartella* ↑ *Faebiobacteria* ↓ **HT group**: *Streptococcus, Altacles, Anaerostipes, Dorea, and Haemophilus* ↑ *Faebiobacteria* ↓	Across-sectional study; small sample size	(86)
28 HT 16 HCs	**HT group**: *Blautia, Roseburia*, *Ruminococcus_torques*_group, *Romboutsia, Dorea, Fusicatenibacter, Eubacterium_hallii*_group genera ↑ *Fecalibacterium, Bacteroides, Prevotella*_9, *Lachnoclostridium* genera↓	Single-center, cross-sectional study; small sample size	(87)
52 GD 45 HCs	**GD group**: *Bacteroidetes, Bifidobacterium*, and *Faecalococcus*↓	Small sample size	(88)
62 GO 18 HCs	**GO group**: *Klebsiella pneumoniae* ↑ in gut microbiota diversity: no significant difference	Small sample size; GO group was distribution; control group was insufficient;	(89)
105 GD 41 HCs	**GD group**: *Actinomycetes* ↑ *Bacteroidetes* ↓ F/B↑	Dietary heretogeneity	(78)
15 GD 14 HCs	**GD group**: *Bacillus*, *Veronococcus, Streptococcus* ↑	Small sample size; impact of dietary drugs	(90)
55 GD 48 HCs	**GD group**: Firmicutes ↓ Bacteroidetes ↑ *Prevotellaceae, Veronicaceae*, and *Prevotella* spp. are closely related to GD patients.	Single-center cross-sectional studies; Small sample size	(91)
45 GD 59 HCs	**GD group**: phylum level: Firmicutes ↓ Bacteroidetes ↑ genus level: *Bacteroides* and *Lactobacillus* ↑ *Blautia*, *Eobacterium*, Anaerobic stick, *Collinsella, Dorea* ↓	Small sample size; uneven distribution between groups	(92)
39 GD 17 HCs	**GD group**: *Bacilli, Lactobacillales, Prevotella, Megamonas, Veillonella* ↑ *Ruminococcus, Rikenellaceae, Alistipes* ↓	Cross-sectional design; 16SrRNA method limitation; small sample size	(93)
33 GO 32 HCs	**GO group**: *Prevotella copri* ↑ *Bacteroides massiliensis*, *Ruminococcus*, *Alistipes shahii*, *Eubacterium hallii*, *Eubacterium ventriosum*, *Marseillibacter massiliensis* ↓	Single-center study; small sample size	(94)

AITD, Autoimmune Thyroid Disease; HT, Hashimoto’s Thyroiditis; GD, Graves’ Disease; HCs, Healthy Controls.

↑ indicates increase bacterial abundances; ↓ indicates decrease bacterial abundances.

Bold text represents study group names.

The table indicates that most AITD patients show reduced gut microbiota diversity, along with changes in some specific genera. It shows reduced beneficial bacteria such as *Bifidobacterium* and *Faecalibacterium* and increased potentially harmful bacteria such as *Bacteroides fragilis*. However, some conflicting findings exist. For instance, Biscarini F and Wen J et al. observed a reduction in Bacteroidetes abundance and an increase in Firmicutes in AITD patients (78). Currently, the trend regarding changes in the genus *Lactobacillus* in clinical samples from AITD patients remains debated. Further studies with rigorous inclusion-exclusion criteria, larger clinical samples, and multicenter collaborations are warranted to validate these findings.

### 3.3 Characteristics of gut microbiota in TN

Multiple clinical studies have consistently demonstrated that patients with TN exhibit gut microbiota dysbiosis, characterized primarily by decreased alpha diversity and changes in the abundance of specific bacterial genera. A Mendelian randomization study on gut microbiota in patients with TN showed that the *Clostridium innocuum* group, *Ruminiclostridium* 5, and *Lachnospira* have protective effects against nontoxic diffuse goiter (NDG). In contrast, Ruminococcaceae UCG002, *Alistipes*, *Methanobrevibacter, Marvinbryantia*, and Ruminococcaceae UCG014 are associated with an increased risk of NDG. *Bifidobacterium* and *Sutterella* have protective effects against nontoxic multinodular goiter (NMG), while the *Ruminococcus gnavus* group and Rikenellaceae RC9 increase the risk of NMG (95). Chen et al. performed 16SrDNA sequencing of fecal microorganisms from 197 participants and found that the alpha-diversity of gut microbiota in TN patients was reduced, with a significant decrease in the relative abundance of butyrate-producing and butyrate-promoting bacteria (96). Li and colleagues conducted a genome-wide association study of the gut microbiome in 196 patients with TN and 283 controls. Their results not only revealed a reduced relative abundance of various butyrate-producing microorganisms but also identified a negative correlation between the L-histidine degradation pathway and thyroid-stimulating hormone (97). This observation indicates that disturbed microbial metabolism is correlated with altered thyroid-related hormone levels, although the specific causal mechanism remains to be further explored. Another study on gut microbiota in TN showed that the abundance of *Neisseria* and *Streptococcus* was increased in the TN patient group, while the abundance of *Butyricimonas* and *Lactobacillus* was decreased (98). These findings confirm that impaired function of butyrate-producing bacteria is a characteristic of gut microbiota dysbiosis in patients with TN.

### 3.4 Characteristics of gut microbiota in TC

As the malignant stage of thyroid lesions, TC also exhibits reduced diversity and altered structure of the gut microbiota. Yu et al. performed 16SrRNA gene sequencing on the gut microbiota samples from 90 TC patients and 90 healthy controls, and observed significantly reduced richness and diversity of the gut microbiota in TC patients. Within the TC group, the relative abundance of *Proteobacteria* was significantly increased, while the abundances of *Prevotella* and Ruminococcaceae were decreased (99). One review concluded that in TC patients, the abundances of *Clostridium*, *Streptococcus*, *Proteus*, and Lachnospiraceae are increased, whereas the abundances of *Lactobacillus*, *Prevotella*, and Ruminococcaceae are decreased (100). These two studies are consistent regarding the changes in *Prevotella* and Ruminococcaceae in the gut microbiota of TC.

However, this conclusion is inconsistent and differs from other studies. A small-sample study of the gut microbiota in TC patients showed that, at the phylum level, Firmicutes and Verrucomicrobia were significantly enriched, while Bacteroidetes were significantly reduced. At the family level, the levels of Ruminococcaceae and Verrucomicrobiaceae were significantly increased, while the abundances of Bacteroidaceae, Prevotellaceae, Porphyromonadaceae, and Alcaligenaceae were significantly decreased (101). Zhu et al. used Mendelian randomization to show a causal relationship between gut microbiota dysbiosis and TC, suggesting that *Butyricimonas* and *Oscillospira* are risk factors for TC, while *Olsenella* and Ruminococcaceae are protective factors (102). Another Mendelian randomization study on gut microbiota in TC found that the abundance of *Actinobacteria* was associated with a reduced risk of TC, while Ruminococcaceae and *Paraprevotella* were associated with an increased risk (103). The differences among these findings may be related to factors such as sample size, dietary structure, and tumor stage. Nevertheless, all these findings indicate that gut microbiota dysbiosis is closely related to TC, and some specific bacteria have a causal relationship with the risk of TC.

### 3.5 Association between gut microbiota and thyroid diseases

The structure of a normal gut microbiota can influence thyroid function by altering the proportions of T cell subsets through immune regulation or by affecting iodine metabolism and trace element absorption via the thyroid-gut axis (104). Furthermore, gut microbiota metabolites, such as short-chain fatty acids and bile acids, can repair the mucosal barrier and exert anti-inflammatory effects by regulating CD4^+^ T cells (105, 106). Gut microbiota modulate thyroid hormone homeostasis primarily via regulation of iodothyronine deiodination and promotion of iodothyronine enterohepatic circulation (107). On the one hand, microbial metabolites suppress intestinal 5-deiodinase activity, thereby altering the local conversion of T4 to T3 and rT3 (108, 109). On the other hand, intestinal bacteria secrete β-glucuronidases and sulfatases to hydrolyze bile-excreted conjugated thyroid hormones. Liberated iodothyronines are subsequently reabsorbed in the gut, sustaining their enterohepatic circulation (110, 111).

Gut dysbiosis attenuates these homeostatic protective effects. Severe dysbiosis can induce immune imbalance and intestinal barrier injury, contributing to thyroid pathogenesis. Multiple cross-sectional clinical studies have revealed that patients with AITD exhibit not only Treg/Th17 imbalance but also markedly elevated serum levels of intestinal permeability markers including zonulin and lipopolysaccharide (LPS) (112). Several interventional studies further demonstrate that gut dysbiosis upregulates zonulin expression, disrupts intestinal epithelial tight junctions, and increases intestinal permeability (113). Impaired intestinal barrier facilitates the translocation of bacterial products and food antigens, which exacerbate thyroid autoimmune responses via molecular mimicry, bystander activation, and epitope spreading (114). Based on these pathological mechanisms, researchers have proposed probiotic supplementation, fecal microbiota transplantation, and trace-element replenishment as potential interventions for patients with thyroid diseases (115, 116). Nevertheless, large-scale clinical studies are still lacking to validate these therapeutic strategies. The Interplay Between Vitamin D and Gut Microbiota.

## 4 The interplay between vitamin D and gut microbiota

### 4.1 Regulatory effects of vitamin D on gut microbiota

Beyond its direct effects on thyroid function and immunity, vitamin D exerts pleiotropic actions on the gut ecosystem, where it regulates microbial composition, diversity, and metabolic function via the VDR pathway. Studies in VDR-knockout mice have shown significant enrichment of Bacteroidetes and Proteobacteria, along with depletion of beneficial butyrate-producing bacteria including Firmicutes, Lactobacillaceae, and Lachnospiraceae (117, 118). A cross-sectional analysis by Luthold et al. involving 150 healthy adults showed a trend of increasing LPS levels as vitamin D levels decreased. In the same study population, the genus *Prevotella* was more abundant in the group with the highest vitamin D intake, while the abundances of *Coprococcus* and *Bifidobacterium* were negatively correlated with vitamin D levels (119). Another clinical study found that vitamin D supplementation significantly increased gut microbial diversity and raised the abundance of beneficial bacteria such as *Akkermansia* and *Bifidobacterium* (120). These two studies are relatively consistent in concluding that vitamin D can increase *Bifidobacterium*. A controlled experiment by Schäffler et al. on patients with Crohn’s disease in clinical remission and healthy individuals showed that after one week of vitamin D supplementation, patients had significant increases in the abundance of *Alistipes*, *Barnesiella*, Porphyromonadaceae, *Roseburia*, *Anaerotruncus*, *Coprococcus*, and unclassified Ruminococcaceae (121). A review also notes that multiple intervention studies suggest that vitamin D can alter microbiota composition, promoting the growth of beneficial bacteria such as Ruminococcaceae, *Akkermansia*, *Faecalibacterium*, and *Coprococcus* (122), echoing Schäffler’s team’s findings. However, the conclusions of the Luthold and Schäffler teams on whether vitamin D supplementation increases *Coprococcus* are inconsistent, possibly due to differences in study populations, vitamin D dosages, and methodologies. A cross-sectional analysis by Thomas et al. of 567 older men indicated that men with higher serum 1,25(OH)_2_D_3_ levels had richer Firmicutes in their gut microbiota and a tendency toward enrichment of butyrate-producing species (123). This aligns with Schäffler’s team’s conclusion that vitamin D increases Firmicutes. Notably, another review proposed that appropriate vitamin D levels could increase Bacteroidetes and decrease Firmicutes (122). Meanwhile, a systematic review found that vitamin D supplementation affects the composition and diversity of Firmicutes, Actinobacteria, and Bacteroidetes, with families such as Veillonellaceae and Oscillospiraceae decreasing as 25(OH)D levels rise or with supplementation (124). These discrepancies may be related to factors such as age, geographic location, study populations’ diets, and inconsistent vitamin D supplementation standards.

Currently, research on the direct regulation of gut microbial metabolites by vitamin D remains limited, and the mechanisms are unclear. A mouse experiment by Nishida et al. found that VDR knockout reduced total bile acid and the primary bile acid chenodeoxycholic acid (CDCA) levels in the liver, feces, and urine, suggesting VDR involvement in CDCA metabolism (125). Chen et al. demonstrated in a high-fat diet mouse model that vitamin D_3_ could modulate gut microbiota, improve intestinal barrier function, and alleviate bile acid metabolism dysfunction in the gut-liver axis (126). Thompson et al. confirmed that secondary bile acids drive bone marrow myelopoiesis dependent on VDR signaling (127). Another experiment indicated that the secondary bile acid lithocholic acid (LCA) requires the intestinal epithelial VDR pathway to upregulate intestinal phosphate absorption (128). These results collectively illustrate the important roles of vitamin D and its receptor, VDR, in bile acid metabolism and related physiological functions. Regarding butyrate metabolism, a recent study by the Feng team in a high-fat model mouse found that vitamin D supplementation increased colonic butyrate production (129). Furthermore, a genome-wide association analysis of gut microbiota and VDR suggested that VDR deficiency reduces gut microbiota diversity and correlates with bile acid and fatty acid indices in the microbiota (15), providing clues to the association among vitamin D, the microbiota, and metabolites.

Based on the above evidence, we can summarize that vitamin D supplementation can increase gut microbiota diversity and elevate the abundance of beneficial bacteria, such as *Akkermansia*, *Bifidobacterium* and Ruminococcaceae. Additionally, vitamin D participates in regulating bile acid metabolism and butyrate production, though the specific mechanisms remain to be elucidated.

### 4.2 Regulatory effect of gut microbiota on vitamin D-VDR

Having explored the significant regulatory role of vitamin D on gut microbiota and its metabolites, we now ask: do gut microbiota and their metabolites in turn affect vitamin D and its receptor? Existing research provides relevant evidence on this question. Studies show that probiotics can directly regulate the expression and activity of the VDR (15). Wu et al. found in a mouse experiment that the probiotic *Lactobacillus* could increase intestinal VDR expression and Paneth cell numbers, thereby inhibiting pathogenic bacterial invasion and inflammatory responses (130). Yoon et al. used a germ-free pig model and found that, compared with the germ-free state, intestinal VDR expression significantly increased after probiotic colonization (131), further supporting the positive regulation of VDR by probiotics.

Moreover, gut microbiota metabolites such as bile acids and butyrate can directly influence vitamin D and VDR. In a mouse experiment, the Kühn team found that cholesterol might enhance the bioavailability of orally administered vitamin D by stimulating bile acid release and increasing bile hydrophobicity (132). Another animal study indicated that bile acids, especially LCA, upregulate CYP27B1 expression *in vitro* and *in vivo*, inducing 1,25(OH)_2_D_3_ production in osteoblasts and renal proximal tubule cells (133). A review reports that bile acids are both key ligands for VDR and regulators of VDR expression (134). Specifically, secondary bile acids can bind specifically to the VDR ligand-binding domain. A study found that adding 2% sodium butyrate to the drinking water increased intestinal VDR expression in mice with colitis and suppressed inflammation (135). Gaschott et al. demonstrated in Caco-2 cells (a human intestinal epithelial cell line) that tributyrin (a triglyceride-bound prodrug of butyric acid) significantly increased VDR mRNA levels and its binding activity (136). Butyrate can also increase nuclear VDR protein content in a time- and dose-dependent manner. Another experiment on renal tubular epithelial cells further showed that butyrate not only induces VDR gene transcription but also stabilizes and activates the VDR protein (137). These results indicate that butyrate can directly upregulate VDR expression and function.

In summary, a clear bidirectional regulatory relationship exists between vitamin D and gut microbiota. Gut microbiota metabolites play a key mediating role in this interaction. They are involved in the generation and activation of vitamin D on one hand, and directly regulate VDR expression and activity on the other, thereby constituting a complex nutrient-microbiota-host axis regulatory network.

## 5 The co-regulating effects of vitamin D and gut microbiota in thyroid diseases

Vitamin D and gut microbiota co-regulate thyroid diseases through immune modulation and intestinal barrier protection (**Figure 2**). Regarding immune regulation, vitamin D, via its receptor VDR, inhibits the differentiation of naïve CD4^+^ T cells into pro-inflammatory Th1 and Th17 cells while promoting their conversion into Th2 and Treg cells (31). As noted in Part 4, vitamin D not only increases the abundance of most butyrate-producing beneficial bacteria but also directly regulates the generation and metabolism of butyrate and bile acids. Butyrate, acting as a histone deacetylase inhibitor, promotes histone acetylation in the Foxp3 gene region, a key transcription factor for Treg cells (138). Primary bile acids, on the other hand, contribute to the differentiation and proliferation of RORγt^+^ Tregs and Foxp3^+^ Tregs (105). Notably, beneficial bacteria, butyrate, and bile acids can, in turn, enhance the expression and activity of vitamin D and VDR, forming a positive feedback loop. This loop further amplifies the regulation of the Th17/Treg balance, thereby effectively alleviating immune dysregulation in thyroid diseases.

**Figure 2:**
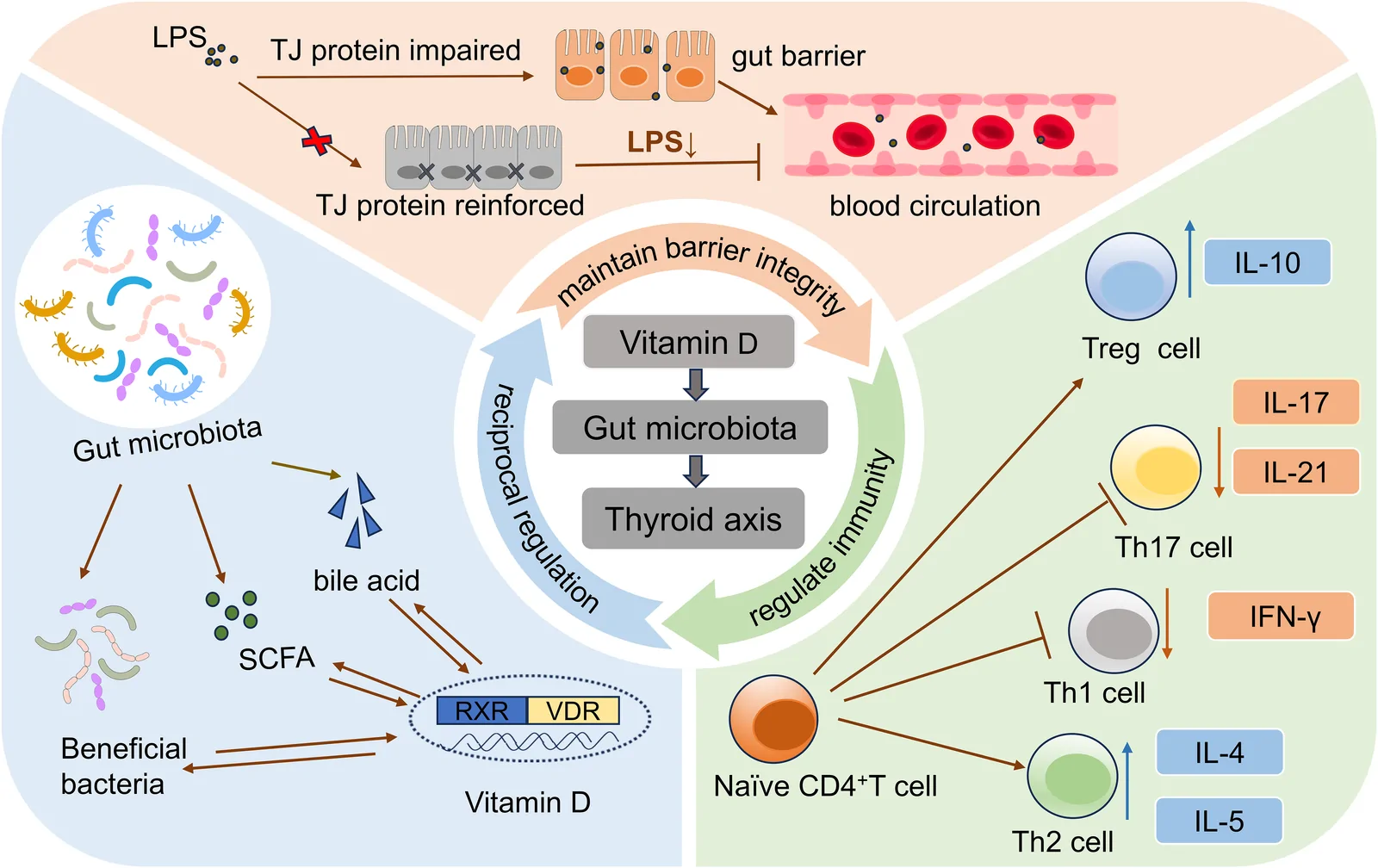
Mechanistic diagram of the vitamin D-gut microbiota-thyroid axis. Vitamin D and gut microbiota have a mutual regulatory effect, jointly affecting the immune system and the intestinal barrier to regulate thyroid function. In the immune system, they can promote the differentiation of naive T cells into Treg cells while inhibiting their differentiation into TH17 cells, thereby regulating the TH17/Treg balance. Regarding the intestinal barrier, they can increase the expression of tight junction proteins to maintain the intestinal barrier and prevent LPS from entering the bloodstream. LPS: Lipopolysaccharide; TJ protein: Tight junction protein; ↑: increase; ↓: decrease.

Impaired intestinal barrier can exacerbate thyroid autoimmune responses, thereby contributing to the pathogenesis of thyroid diseases (112). In terms of maintaining the intestinal barrier, probiotics, the microbial metabolite butyrate, and vitamin D also exhibit synergistic effects. A study using a mouse colitis model showed that combined administration of butyrate and vitamin D enhanced expression of defensive cytokines and antimicrobial peptides in the cecum and reduced zonulin and claudin-2 levels in the mucosal layer (139). Claudin-2 primarily functions to form selective cation channels, particularly with high sodium ion permeability (140). Its downregulation suggests reduced intestinal permeability, indicating that the combined action of butyrate and vitamin D helps maintain intestinal barrier integrity. Butyrate, as the primary energy source for colonic epithelial cells, also promotes epithelial cell proliferation and repair, thereby further strengthening the intestinal barrier (141). Additionally, articles note that some probiotics can stimulate goblet cells to produce mucus, which contributes to mucus layer formation and further enhances the intestinal barrier (142).

In summary, vitamin D and gut microbiota synergistically modulate the pathological progression of thyroid diseases. On the one hand, vitamin D, together with gut microbiota and their metabolites, orchestrates Th17/Treg cell balance to suppress thyroid-targeted autoimmune responses. On the other hand, these two factors cooperate to preserve intestinal mucosal architecture, reduce intestinal leakage, and mitigate auto-inflammatory responses. Furthermore, vitamin D and gut microbiota-derived metabolites mutually regulate one another to form a positive-feedback loop that amplifies the aforementioned protective effects. Based on the above research evidence, this coordinated regulation mode may constitute an important mechanistic basis for the vitamin D-gut microbiota-thyroid regulatory axis.

## 6 Future perspectives and conclusions

Current evidence confirms that vitamin D insufficiency and gut dysbiosis are prevalent in patients with thyroid disorders. But most clinical and basic research mainly focuses on their respective impacts on thyroid diseases. And most studies of vitamin D in thyroid diseases are limited to clinical samples. The research on the pathogenesis of vitamin D in thyroid diseases has not been explored in depth. Most of them only focus on immune regulation. The current situation of research on the role of gut microbiota in thyroid diseases also has the same limitation. Vitamin D and gut microbiota exert mutual regulatory effects, but preclinical and clinical studies exploring their combined interventions in thyroid diseases remain scarce. Therefore, to fill this gap, in this review, we have summarized the basic and clinical research on vitamin D and gut microbiota in thyroid diseases, as well as the mutual regulatory functions of vitamin D and gut microbiota. We propose the novel concept of the vitamin D-gut microbiota-thyroid axis. This axis represents a reciprocal regulatory loop, in which vitamin D maintains gut microbiota homeostasis and intestinal barrier function, while gut microbiota and its metabolites modulate vitamin D bioavailability and signaling, ultimately influencing thyroid immune tolerance and function. Disruption of this axis, such as vitamin D insufficiency combined with gut dysbiosis, may trigger chronic low-grade inflammation and autoimmune responses in the thyroid, contributing to the onset and progression of thyroid diseases.

The proposal of this axis holds profound significance for thyroid disease research. However, studies on the vitamin D-gut microbiota-thyroid axis still face several critical gaps. Current evidence is largely correlative, lacking direct causal validation from pre-clinical models such as germ-free animals and fecal microbiota transplantation studies. The cell-type-specific mechanisms through which vitamin D and gut microbiota regulate thyroid function remain poorly defined. Strategies targeting the whole “vitamin D-gut microbiota-thyroid axis” are still hypothetical. Clinical verification of such combined interventions is currently lacking. The intervention effects of vitamin D supplementation alone differ greatly among individuals. Precision-nutrition regimens for vitamin D tailored to genetic background and gut microbiota signatures also remain under active exploration. Addressing these issues will not only deepen our understanding of the pathogenesis of thyroid diseases but also provide a theoretical foundation for the development of novel multi-target therapeutic strategies. For example, vitamin D supplementation combined with probiotics or fecal microbiota transplantation holds promise for improving clinical outcomes in patients with thyroid disorders by restoring the homeostasis of this axis. So future efforts should involve large-scale, multicenter, prospective clinical studies to further clarify the specific molecular mechanisms of the vitamin D-gut microbiota-thyroid axis to fill these limitations.

On the whole, vitamin D and gut microbiota offer new directions for the prevention and treatment of thyroid diseases. Integrating the concept of the vitamin D-gut microbiota-thyroid axis with precision-nutrition perspectives holds promise for advancing thyroid disease management toward multi-targeted and personalized intervention.
